# Clinical significance and correlation of PD-L1, B7-H3, B7-H4, and TILs in pancreatic cancer

**DOI:** 10.1186/s12885-022-09639-5

**Published:** 2022-05-27

**Authors:** Jiayue Yang, Zhen Tian, Han Gao, Fan Xiong, Cuiping Cao, Jiaojiao Yu, Wei Shi, Qiang Zhan, Cheng Yang

**Affiliations:** 1 Department of Endocrinology, The Affiliated Wuxi People’s Hospital of Nanjing Medical University, Wuxi, Jiangsu Province, 214023 China; 2 Department of Clinical Laboratory, The Affiliated Wuxi People’s Hospital of Nanjing Medical University, Wuxi, Jiangsu Province, 214023 China; 3 Wuxi School of Medicine, Jiangnan University, Wuxi, 214122 China; 4 Department of Gastroenterology, The Affiliated Wuxi People’s Hospital of Nanjing Medical University, No. 299 Qing Yang Road, Wuxi, Jiangsu Province, 214023 China

**Keywords:** PD-L1, B7-H3, B7-H4, Tumor immunity, Pancreatic cancer

## Abstract

**Background:**

B7 molecules play significant roles in regulating tumor immunity, but their expression patterns and immuno-biological correlations in pancreatic cancer (PaCa) have not been fully discussed.

**Methods:**

RNA-sequencing data of B7 molecules of PaCa samples in the Cancer Genome Atlas (TCGA) dataset was downloaded from the UCSC Xena to assess the expression, correlation, and mutation of the B7 family in PaCa. Next, two PaCa tissue microarrays (TMAs, Cat. HPanA150CS02 and HPanA120Su02) were obtained from Outdo BioTech (Shanghai, China). To detect the expression levels of PD-L1, B7-H3 and B7-H4, immunohistochemistry (IHC) staining was performed on these TMAs.

**Results:**

Most B7 molecules, including B7–1, B7–2, PD-L1, B7-DC, B7-H2, and B7-H5 exhibited similar expression patterns, but B7-H3, B7-H4, B7-H6, and B7-H7 showed outlier expression patterns compared with other B7 molecules. Besides, B7 molecules were genetically stable and exhibited low alteration frequency. IHC staining indicated PD-L1, B7-H3, and B7-H4 were up-regulated in PaCa tissues and showed uncorrelated expression patterns. Furthermore, high expression of PD-L1 and B7-H3 indicated poor-differentiated grades in PaCa. PD-L1 was positively, but B7-H4 was negatively correlated with CD8+ TILs infiltration in PaCa. Moreover, combined PD-L1 and B7-H4 expression was a novel subtyping strategy in PaCa, namely patients with both high PD-L1 and B7-H4 expression exhibited decreased CD8+ TILs infiltration in tumor tissues.

**Conclusion:**

Overall, we systemically analyzed the expression patterns of B7 molecules and proposed a novel subtyping strategy in PaCa. Patients with both high PD-L1 and B7-H4 expression exhibited the immuno-cold phenotype, which may be not suitable for immunotherapy.

**Supplementary Information:**

The online version contains supplementary material available at 10.1186/s12885-022-09639-5.

## Background

Pancreatic cancer (PaCa) is a highly malignant tumor of the digestive system, about 90% of which is ductal adenocarcinoma originating from the ductal epithelium. PaCa is difficult to diagnose and treat, and its morbidity and mortality have increased significantly in recent years [1]. According to the newest statistic data published by Sung et al., there will be about 496,000 new cases of PaCa and 466,000 cancer-causing deaths in 2020 [2]. Due to the dreadful aggressiveness and the difficulty of early detection, PaCa is one of the malignant tumors with the worst prognosis and accounts for almost as many deaths as new cases [2]. In contrast to most other cancer types, improvement of the therapeutic options and prognosis over the last decades has been quietly limited [3, 4].

Immune checkpoint blockade (ICB) is inspiring immunotherapy transforming the standard of treatment for several advanced tumors [5]. Representative immune checkpoint inhibitors are monoclonal antibodies that interfere with the interaction between PD-1 and PD-L1 inhibitory proteins expressed on the surface of T cells and tumor cells, respectively. Unfortunately, the response rate to ICB monotherapy remains low in PaCa [6, 7]. A recent phase 2 clinical trial combining durvalumab plus tremelimumab exhibited a mere objective response (OR) rate of 3.1% [8]. Therefore, effective immune-related biomarkers are urgent to identify patients who will benefit from ICB and to stratify patients into optimal immunotherapy strategies.

Tumor mutation burden (TMB) and T cell inflamed gene expression profile are useful biomarkers for response to ICB in cancers [9]. In clinical practice, the detection of PD-L1 has been considered a predictive biomarker for anti-PD-1/PD-L1 therapies. Patients with high PD-L1 expression tended to be highly responsive to PD-1/PD-L1 blockade [10, 11]. In addition to PD-L1, B7-H3 and B7-H4 are highly expressed in most tumor tissues, making them attractive candidate immunotherapeutic targets and biomarkers [12, 13]. Furthermore, the combination of PD-L1 and B7-H4 expression is a novel classification strategy in glioma, namely the high B7-H4-expressed samples could be considered super-cold gliomas with significantly deficient in tumor-infiltrating lymphocytes (TILs) [14]. However, the expression patterns of PD-L1, B7-H3, and B7-H4 in PaCa have not been fully discussed.

Given the evidence and the clinical need to explore immune-related biomarkers to stratify patients into optimal immunotherapy strategies, the current study aimed to systematically characterize the expression patterns and immuno-biological correlations of B7 family members, especially PD-L1, B7-H3, and B7-H4. Besides, based on the expression levels of PD-L1 and B7-H4, we proposed a novel subtyping strategy in PaCa, namely patients with both high PD-L1 and B7-H4 expression exhibited the immuno-cold phenotype, which may be not suitable for immunotherapy.

## Methods

### Acquisition of TCGA data

Normalized RNA-sequencing data of PaCa samples in the Cancer Genome Atlas (TCGA) dataset was obtained from the UCSC Xena (https://xenabrowser.net/datapages/) or the cBioPortal portal (http://www.cbioportal.org/datasets) [15]. Patients with missing or insufficient data were excluded from this research. The genetic alteration data of B7 molecules was downloaded from the cBioPortal portal.

### Clinical specimens

Two PaCa tissue microarrays (TMAs, Cat. HPanA150CS02 and HPanA120Su02) were obtained from Outdo BioTech (Shanghai, China). The HPanA150CS02 TMA contained 78 PaCa tissues and 72 paired adjacent tissues, which was used as the exploring cohort for the detection of B7 molecules expression and the training cohort for the PD-L1/B7-H4-based stratified strategy. The HPanA120Su02 TMA contained 66 PaCa tissues and 54 paired adjacent tissues, which was used as the validated cohort for the PD-L1/B7-H4-based stratified strategy. Detailed clinico-pathological characteristics of the cohorts were also provided by Outdo BioTech (Shanghai, China). Ethical approval for the study of the TMAs was granted by the Clinical Research Ethics Committee, Outdo BioTech (Shanghai, China).

### Immunohistochemistry

Immunohistochemistry (IHC) staining was performed on these TMAs of PaCa tissues according to the standardized procedure. EDTA was used for antigen retrieval, and the primary antibodies were incubated overnight at 4 °C. The primary antibodies used in the research were as follows: anti-PD-L1 (1:100 dilution, Cat. ab237726, clone: CAL10, Abcam, Cambridge, UK), anti-B7-H3 (1:8000 dilution, Cat. ab219648, clone: EPR20115, Abcam, Cambridge, UK), anti-B7-H4 (1:50 dilution, Cat. ab252438, clone: EPR23665–20, Abcam, Cambridge, UK) and anti-CD8 (Ready-to-use, Cat. PA067, clone: 457F6F8, Abcarta, Suzhou, China). Notably, staining data for PD-L1 and CD8 of the HPanA120Su02 TMA was provided by Outdo BioTech (Shanghai, China). A total of 9 samples that fall off the HPanA120Su02 TMA during the IHC staining for B7-H4. Thus, 57 samples in the HPanA120Su02 TMA were used for further analysis. Antibody staining was visualized using diaminobenzidine and hematoxylin counterstain, and stained TMAs were scanned using Aperio Digital Pathology Slide Scanners.

### Manual quantification of IHC

The results of IHC were evaluated by two independent pathologists using an established semiquantitative approach that assessed the immune-reactivity score (IRS) [16, 17]. For semi-quantitative evaluation of PD-L1, B7-H3, and B7-H4 staining, the percentage of positively stained cells was scored as 0–4: 0 (< 1%), 1 (1–5%), 2 (6–25%), 3 (26–50%) and 4 (> 50%). The staining intensity was scored as 0–3: 0 (negative), 1 (weak), 2 (moderate) and 3 (strong). IRS equals the percentages of positive cells multiplied by staining intensity. Samples with IRS ≤ 2 were deemed to be the low expression and IRS > 2 were deemed to be the high expression. For semi-quantitative evaluation of CD8 staining, the proportion of CD8+ immune cells was assessed by estimating the percentage of cells with strong intensity of membrane staining in the stroma cells. The HPanA150CS02 TMA: the consistency of PD-L1, B7-H3, B7-H4, and CD8 were 74/78 (94.87%), 76/78 (97.43%), 75/78 (96.15%), and 73/78 (93.59%), respectively. The HPanA120Su02 TMA: the consistency of PD-L1, B7-H4, and CD8 were 55/57 (96.49%), 54/57 (94.74%), and 55/57 (96.49%), respectively. Disputed samples were adjudicated by a third senior pathologist.

### Statistical analysis

R 4.1.0, SPSS 26.0, and GraphPad Prism 6.0 were applied as the main tools for the statistical analysis and figures exhibition. Differences between the two groups were analyzed using Student’s t-test or Mann-Whitney test for continuous variables and the chi-square test for categorical variables. Differences between multiple groups were analyzed using one-way ANOVA test or Kruskal-Wallis analysis for continuous variables. Correlation analysis was evaluated by Pearson correlation analysis. Log-rank test was conducted to evaluate the difference between the survival curves. For all statistical analyses, *P* ≤ 0.05 was deemed to be statistically significant.

## Results

### Expression, correlation, and mutation of B7 family in PaCa

**Fig. 1:**
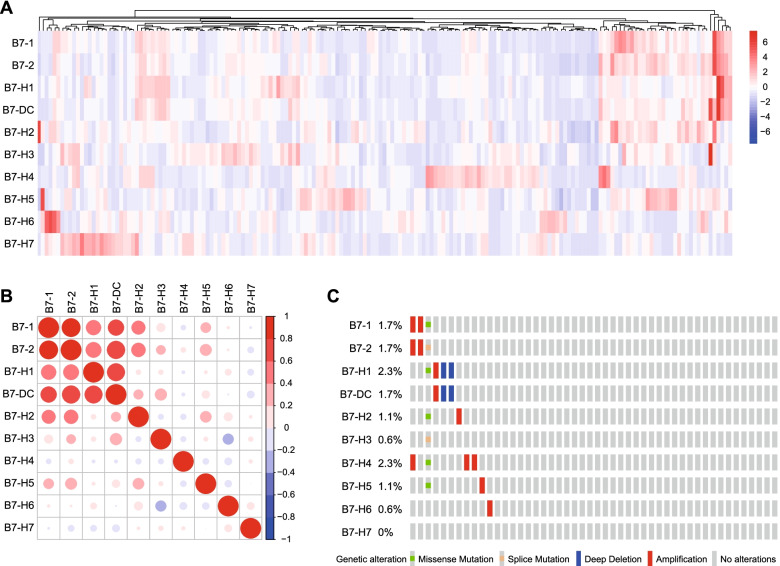
Expression, correlation and mutation of B7 family in PaCa. **A** Expression patterns of B7 molecules in PaCa in the TCGA dataset were summarized in the heatmap. Red: high expression, blue: low expression. **B** Correlations of B7 molecules in PaCa in the TCGA dataset were summarized in the heatmap. The color reveals the Pearson correlation coefficient. **C** OncoPrint in the cBioPortal database exhibited the proportion and distribution of specimens with genetic alterations in B7 molecules. Gene expression and genetic alteration data were obtained from the cBioPortal portal (http://www.cbioportal.org/datasets)

Given the significant role of B7 molecules in tumor immunity, we first systemically explored their expression patterns in PaCa. As exhibited in Fig. 1, the mRNA levels of B7–1, B7–2, PD-L1, B7-DC, B7-H2, and B7-H5 exhibited similar expression pattern, which was highly correlated with each other, but B7-H3, B7-H4, B7-H6, and B7-H7 mRNA levels showed outlier expression patterns compared with other B7 molecules (Fig. 1A, B). Besides, genetic alteration analysis indicated that B7 molecules were genetically stable with low alteration frequency a the scale of 0.6 to 2.3% (Fig. 1C).

**Fig. 2:**
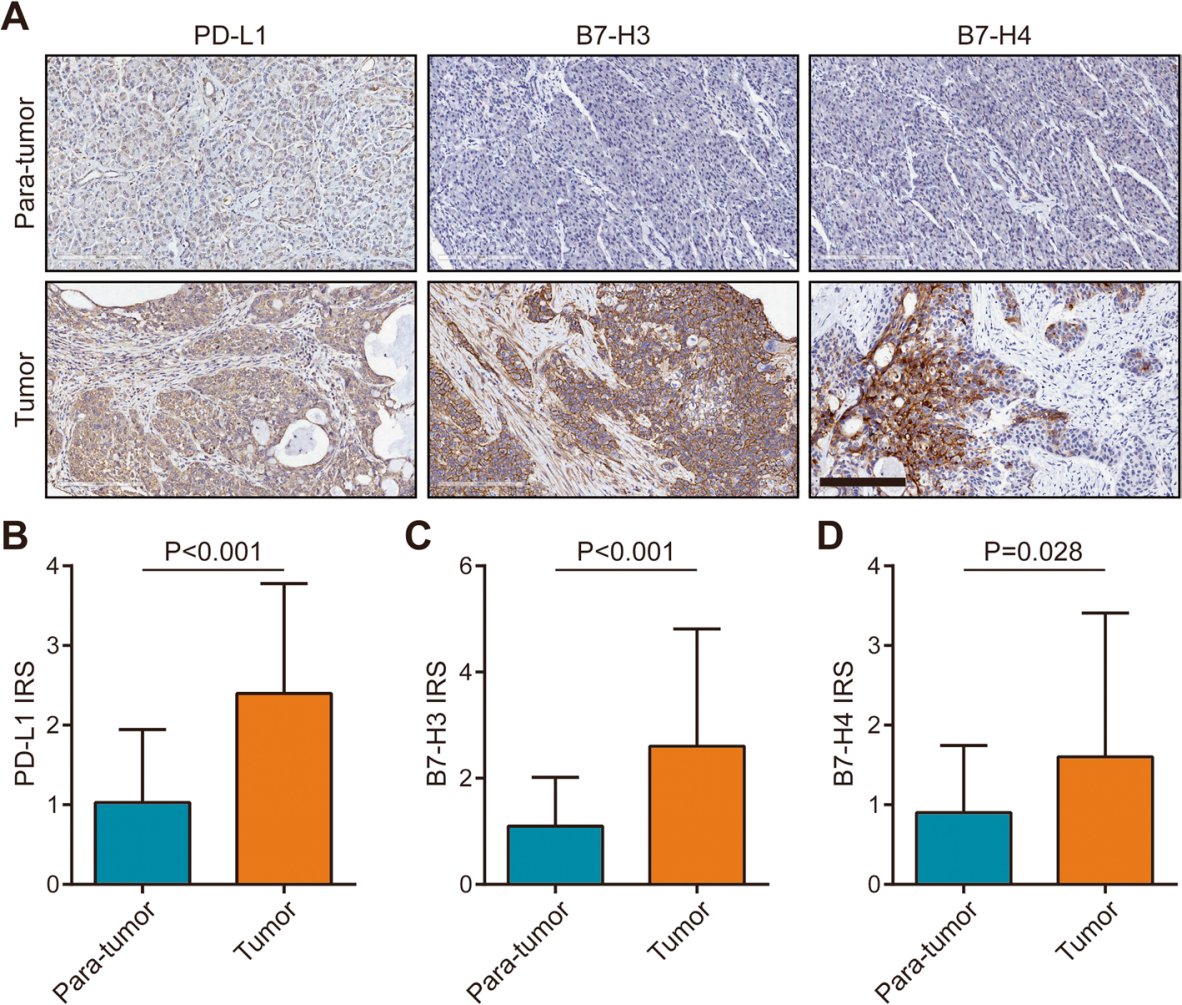
Expression levels of PD-L1, B7-H3, and B7-H4 in PaCa tissues. **A** Representative microphotographs revealed these molecules expression in para-tumor tissues and tumor tissues using IHC staining. Brown, PD-L1, B7-H3, or B7-H4. Blue, haematoxylin. Bar = 200 μm. **B** The expression of PD-L1 in tumor (*n* = 78) and para-tumor (*n* = 72) tissues. **C** The expression of B7-H3 in tumor (*n* = 78) and para-tumor (*n* = 72) tissues. **D** The expression of B7-H4 in tumor (*n* = 78) and para-tumor (*n* = 72) tissues

We next validated the protein expression patterns of B7 molecules in PaCa using IHC staining. Considering the similar expression patterns of B7–1, B7–2, PD-L1, B7-DC, B7-H2, and B7-H5, we selected PD-L1 among these molecules for further validation. Besides, B7-H3 and B7-H4 were also selected for validation due to their relatively clear functions compared with B7-H6 and B7-H7. After analysis of IHC staining, we found that PD-L1, B7-H3, and B7-H4 proteins were all up-regulated in PaCa tissues compared with para-tumor tissues (Fig. 2A-D). Moreover, similar to the results of the TCGA dataset, these three molecules were not correlated with each other (Fig. S1A-C). Overall, PD-L1, B7-H3, and B7-H4 were overexpressed in PaCa and showed uncorrelated expression patterns.

### Association between expression levels of B7 molecules and clinic-pathological parameters in PaCa

**Fig. 3:**
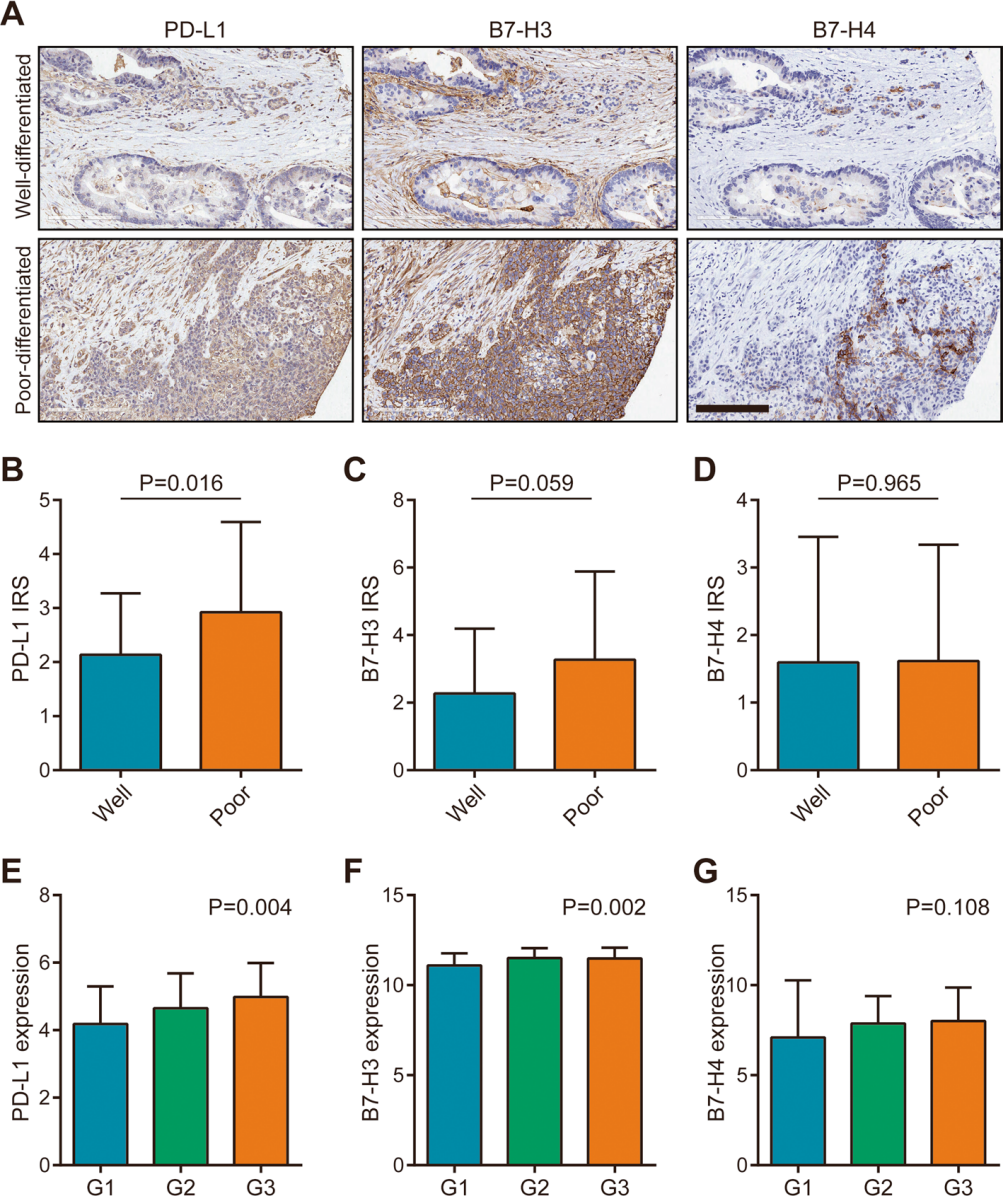
Expression levels of PD-L1, B7-H3, and B7-H4 in PaCa tissues with various differentiations. **A** Representative microphotographs revealed these molecules expression in well-differentiated tissues and poor-differentiated tissues using IHC staining. Brown, PD-L1, B7-H3, or B7-H4. Blue, haematoxylin. Bar = 200 μm. **B** The expression of PD-L1 in variously differentiated (well: *n* = 56, poor: *n* = 26) PaCa tissues. **C** The expression of B7-H3 in variously differentiated (well: *n* = 56, poor: *n* = 26) PaCa tissues. **D** The expression of B7-H4 in variously differentiated (well: *n* = 56, poor: *n* = 26) PaCa tissues. **E** The expression of PD-L1 in variously differentiated (G1: *n* = 31, G2: *n* = 95, G3:*n* = 48) PaCa tissues in the TCGA dataset. **F** The expression of B7-H3 in variously differentiated (G1: *n* = 31, G2: *n* = 95, G3: *n* = 48) PaCa tissues in the TCGA dataset. **G** The expression of B7-H4 in variously differentiated (G1: *n* = 31, G2: *n* = 95, G3:*n* = 48) PaCa tissues in the TCGA dataset

Subsequently, we analyzed the association between expression levels of B7 molecules and clinic-pathological parameters. As shown in Table 1, PD-L1 and B7-H3 protein expression levels were not associated with age, gender, T stage, N stage, M stage, and clinical stage, but associated with tumor differentiation (Table 1). However, B7-H4 protein expression level was not associated with any clinic-pathological parameters in the cohort (Table 1). We next compared the expression levels of PD-L1, B7-H3, and B7-H4 in tumor tissues with various differentiated grades. The results showed that PD-L1 and B7-H3 proteins were up-regulated in poor-differentiated tissues, but B7-H4 protein expression was similar in well- and poor-differentiated tissues (Fig. 3A-D). Moreover, the mRNA data from the TCGA dataset was also in accordance with the current results (Fig. 3E-G). Due to the deficiency of survival data in the current cohort, we utilized the survival data from the TCGA dataset to assess the prognostic values of these three B7 molecules. However, the results showed that mRNA expression of single B7 molecules had limited prognostic value (Fig. S2A-C). Taken together, these results suggested that high expression of PD-L1 and B7-H3 indicated poor-differentiated grades in PaCa.

**Table 1:** Association between B7 molecules expression and clinic-pathological parameters in PaCa

Parameters		n	PD-L1		χ^2^ value	*P* value	B7-H3		χ^2^ value	*P* value	B7-H4		χ^2^ value	*P* value
			low	high			low	high			low	high		
Age	Female	34	21	13	0.378	0.539	22	12	0.063	0.801	26	8	0.001	0.977
	Male	42	23	19			26	16			32	10		
Gender	≤60	28	15	13	0.527	0.468	16	12	0.603	0.437	22	6	0.204	0.652
	> 60	50	31	19			33	17			37	13		
Differentiation^a^	Well	52	35	17	4.478	0.034	37	15	4.638	0.031	41	11	0.870	0.351
	Poor	26	11	15			12	14			18	8		
T stage	≤3 cm	35	18	17	1.494	0.222	20	15	0.876	0.349	27	8	0.078	0.780
	> 3 cm	43	28	15			29	14			32	11		
N stage	N0	49	32	17	1.576	0.209	29	20	2.857	0.091	39	10	0.198	0.656
	N1	24	12	12			19	5			18	6		
M stage^b^	M0	71	42	29	/	1.000	45	26	0.106	0.745	54	17	/	1.000
	M1	7	4	3			4	3			5	2		
Clinical stage	1	42	27	15	0.731	0.393	27	15	0.003	0.954	34	8	0.713	0.398
	2–4	33	18	15			21	12			24	9		

^a^ Well differentiation: Grade I, II and I-II; Poor differentiation: Grade II-III, III

^b^ Checked by the modified chi-square test due to the cases less than 5

### Correlation between B7 molecules and TILs in PaCa

**Fig. 4:**
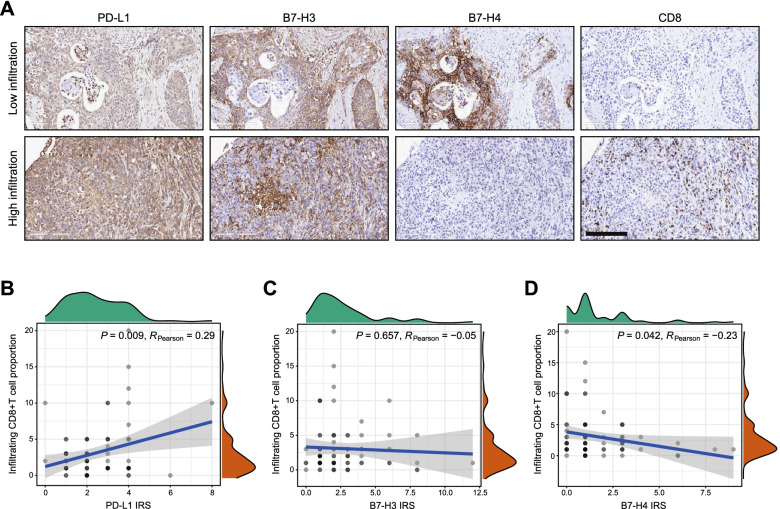
Correlations between PD-L1, B7-H3 as well as B7-H4 expression and CD8+ TILs infiltration in PaCa tissues. **A** Representative microphotographs revealed these molecules expression in PaCa tissues with low and high CD8+ TILs infiltration using IHC staining. Brown, PD-L1, B7-H3, B7-H4, or CD8. Blue, haematoxylin. Bar = 200 μm. Low CD8+ TILs infiltration: proportion < 5%, how CD8+ TILs infiltration: proportion ≥ 5%. **B** Correlation between PD-L1 expression and CD8+ TILs levels in PaCa tissues. **C** Correlation between B7-H3 expression and CD8+ TILs levels in PaCa tissues. **D** Correlation between B7-H4 expression and CD8+ TILs levels in PaCa tissues

Previous research tried to explore the correlation between B7 molecules and TILs in multiple cancers [18–20]. Thus, we also checked the correlation between PD-L1, B7-H3 as well as B7-H4 protein expression and TILs levels in PaCa. Figure 4A showed representative staining for PD-L1, B7-H3 and B7-H4 in the tissues with low and high CD8+ TILs infiltration. The results indicated that PD-L1 was positively, but B7-H4 was negatively correlated with CD8+ TILs infiltration in PaCa (Fig. 4B, D). Besides, B7-H3 was not correlated with CD8+ TILs infiltration in PaCa (Fig. 4C). To conclude, PD-L1 and B7-H4 could be used as indicators for assessing the infiltration level of CD8+ TILs.

### PD-L1/B7-H4 stratify infiltrating levels of TILs in PaCa

**Fig. 5:**
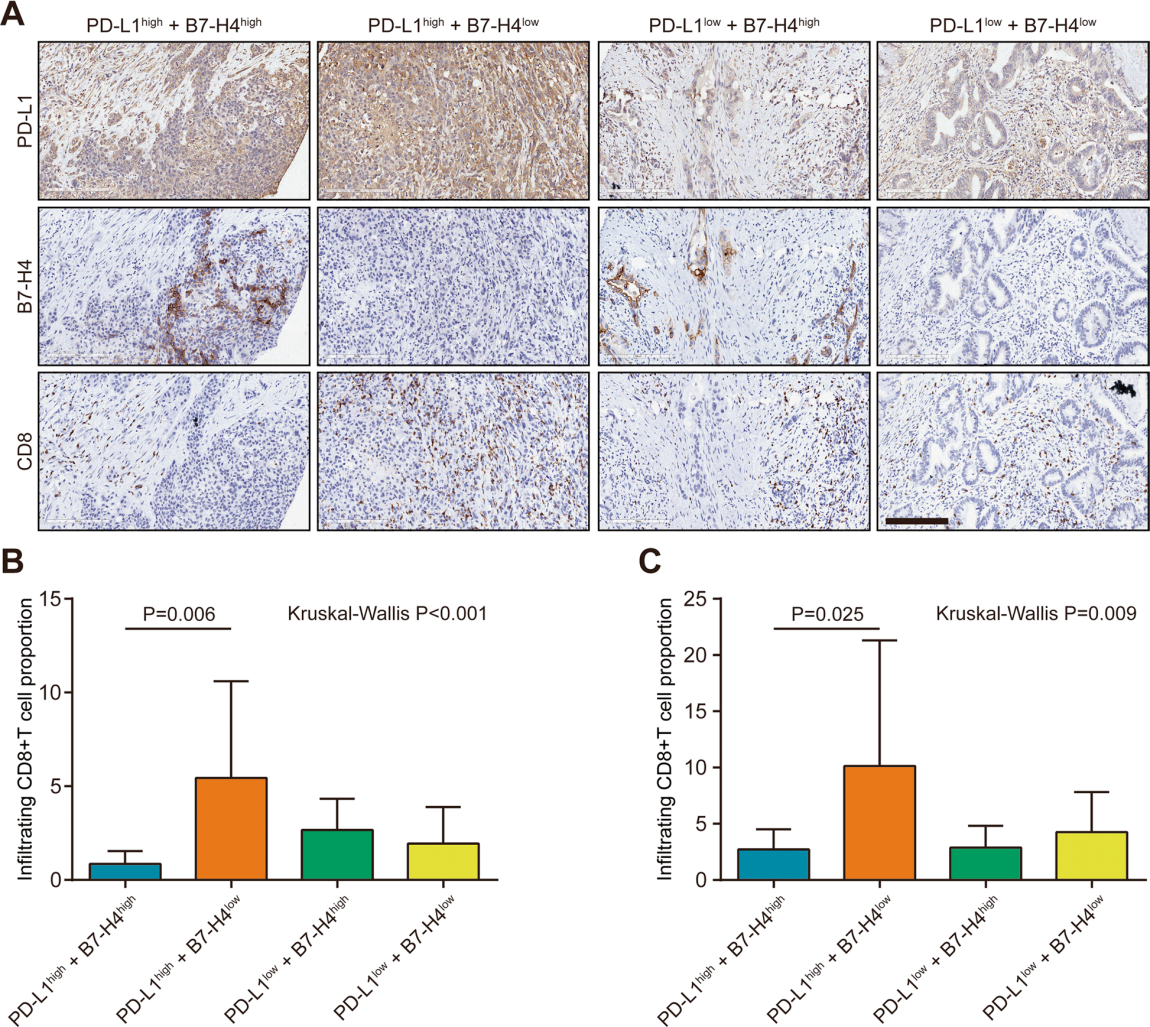
The predictive value of PD-L1/B7-H4 classifier in predicting CD8+ TILs levels in PaCa. **A** Representative microphotographs revealed PD-L1 and B7-H4 expression as well as CD8+ TILs levels in PD-L1^high^-B7-H4^high^, PD-L1^high^-B7-H4^low^, PD-L1^low^-B7-H4^high^, and PD-L1^low^-B7-H4^low^ groups using IHC staining. Brown, PD-L1, B7-H4, or CD8. Blue, haematoxylin. Bar = 200 μm. **B** The levels of CD8+ TILs in these groups in the HPanA150CS02 cohort. PD-L1^high^-B7-H4^high^: *n* = 7, PD-L1^high^-B7-H4^low^: *n* = 25; PD-L1^low^-B7-H4^high^: *n* = 12, PD-L1^low^-B7-H4^low^: *n* = 34. **C** The levels of CD8+ TILs in these groups in the HPanA120Su02 cohort. PD-L1^high^-B7-H4^high^: *n* = 8, PD-L1^high^-B7-H4^low^: *n* = 24; PD-L1^low^-B7-H4^high^: *n* = 17, PD-L1^low^-B7-H4^low^: *n* = 8

Given the correlation between PD-L1 and B7-H4 protein expression and TILs infiltration, we next tried to develop a novel subtyping strategy to estimate immuno-phenotype in PaCa. We divided PaCa patients into four subgroups, namely PD-L1^high^-B7-H4^high^, PD-L1^high^-B7-H4^low^, PD-L1^low^-B7-H4^high^, and PD-L1^low^-B7-H4^low^ groups (Fig. 5A). According to previous research, high PD-L1 expression was correlated with increased CD8+ TILs infiltration and was the reliable biomarker for immuno-hot tumors [21]. However, the results showed that decreased CD8+ TILs infiltration in the PD-L1^high^-B7-H4^high^ group compared with the PD-L1^high^-B7-H4^low^ group (Fig. 5B). The HPanA120Su02 TMA contained 66 PaCa tissues was used as the validated cohort. In the HPanA120Su02 cohort, PD-L1 was positively correlated with CD8+ TILs infiltration but B7-H4 was not correlated with CD8+ TILs infiltration in PaCa (Fig. S3A-C). The result of the HPanA120Su02 cohort was in accordance with the current result of the HPanA150CS02 cohort (Fig. 5C). Overall, these data suggested PaCa patients with both high PD-L1 and B7-H4 expression exhibited the immuno-cold phenotype, which may be not suitable for immunotherapy.

## Discussion

Increasing numbers of clues suggest that the dys-regulated expression of B7 molecules results in the suppressive tumor microenvironment (TME) and immune escape [22]. The B7 family consists of 10 members, namely B7–1 (CD80), B7–2 (CD86), B7-H1 (CD274/PD-L1), B7-DC (PDCD1LG2), B7-H2 (ICOSLG), B7-H3 (CD276), B7-H4 (VTCN1), B7-H5 (VSIR), B7-H6 (NCR3LG1), and B7-H7 (HHLA2) [23]. In this research, we first systemically explored the expression patterns of these B7 molecules in PaCa. B7–1, B7–2, PD-L1, B7-DC, B7-H2, and B7-H5 exhibited similar expression pattern, which was highly correlated with each other, but B7-H3, B7-H4, B7-H6, and B7-H7 showed outlier expression patterns compared with other B7 molecules. Besides, B7 molecules were genetically stable with low alteration frequency in PaCa, suggesting that genetic alterations might not be critical threats to human cancers.

In recent years, the dominating roles of the B7 molecules in regulating tumor immunity have been widely concerned, especially PD-L1, B7-H3, and B7-H4, which have become hot research objects [19, 24]. To study their respective roles in the TME, the expression profiles and biological roles of PD-L1, B7-H3, and B7-H4 in cancers have been well-concluded. For example, Qiu et al. analyzed the expression and prognostic values of these B7 molecules and found that B7-H3 was a negative prognostic indicator in small cell lung cancer [25]. In this study, PD-L1, B7-H3, and B7-H4 were applied as research objects to be focused on, and their expression, clinical significance, and correlation with TILs in PaCa were explored. The critical results of the current study may provide a promising scientific basis for clinical diagnosis and therapeutic assessment.

In PaCa samples in the current cohort, we found that PD-L1, B7-H3, and B7-H4 were all overexpressed in PaCa tissues compared with para-tumor tissues, which was in accordance with other studies [26–28]. Besides, the protein levels of these three molecules were not correlated with each other as well. Moreover, PD-L1 and B7-H3 were up-regulated in poor-differentiated tissues, but B7-H4 expression was similar in well- and poor-differentiated tissues. However, survival analysis showed that single B7 molecules had limited prognostic value. Furthermore, we also checked the correlation between these B7 molecules expression and TILs levels in PaCa in this research. PD-L1 was positively, and B7-H4 was negatively correlated with CD8+ TILs infiltration, but B7-H3 was not correlated with CD8+ TILs infiltration in PaCa.

Growing numbers of research have reported that mutually-exclusive or co-expressed patterns of B7 molecules could predict inflamed or non-inflamed TME in multiple human cancers [14, 29]. For example, B7-H4 is negatively correlated with PD-L1 and identifies immuno-cold tumors in glioma [14]. Considering the various correlations between PD-L1 and B7-H4 expression and TILs infiltration, we tried to develop a novel subtyping strategy to estimate immuno-phenotype in PaCa based on PD-L1 and B7-H4 expression. High PD-L1 expression has been considered a reliable biomarker for high response to immunotherapy due to its high correlation with effector TILs [21, 30–32]. First, PD-L1 was not correlated with B7-H4 expression in PaCa in the current cohort. Importantly, the results of our study indicated decreased CD8+ TILs infiltration in PaCa patients with both high PD-L1 and B7-H4 expression, and the result was also validated in another independent HPanA120Su02 cohort. Collectively, PaCa patients with both high PD-L1 and B7-H4 might be dismissed from the immunotherapy in clinical practice.

The current research also had several shortcomings. First of all, the expression, correlation, and mutation of B7 family in PaCa were investigated using the public TCGA cohort, which were not validated using the in-house cohort. In addition, there may be a certain discrepancy between mRNA and protein level expression, and RNA-sequencing data can not distinguish the cell types. Furthermore, although we proposed a novel subtyping strategy in PaCa, it was not validated in any immunotherapy cohort in clinical practice.

## Conclusion

In the current research, we systemically analyzed the expression patterns of B7 molecules and found a significant immuno-biological correlation of PD-L1 and B7-H4 in PaCa. Based on the expression status of PD-L1 and B7-H4, we proposed a novel subtyping strategy in PaCa to assess immune cells infiltration. PaCa patients with both high PD-L1 and B7-H4 expression exhibited the immuno-cold phenotype, which should be excluded from immunotherapeutic dominance.

## Supplementary Information

**Additional file 1: Fig. S1.** Correlations of these three B7 molecules in PaCa tissues. (A) Correlation between PD-L1 and B7-H3 expression in PaCa tissues. (B) Correlation between PD-L1 and B7-H4 expression in PaCa tissues. (C) Correlation between B7-H3 and B7-H4 expression in PaCa tissues.

**Additional file 2: Fig. S2.** Survival plots of these three B7 molecules in PaCa patients. (A) Prognostic value of *PD-L1* mRNA expression in PaCa patients. (B) Prognostic value of *B7-H3* mRNA expression in PaCa patients. (C) Prognostic value of *B7-H4* mRNA expression in PaCa patients. Gene expression and survival data were obtained from UCSC Xena (https://xenabrowser.net/datapages/).

**Additional file 3: Fig. S3.** Correlations between PD-L1 & B7-H4 expression and CD8+ TILs infiltration in PaCa tissues (HPanA120Su02 cohort). (A) Correlation between PD-L1 expression and CD8+ TILs levels in PaCa tissues. (B) Correlation between B7-H4 expression and CD8+ TILs levels in PaCa tissues.

## Data Availability

All data are included in the article.

## Abbreviations

PaCa: Pancreatic cancer
ICB: Immune checkpoint blockade
OR: Objective response
TMB: Tumor mutation burden
TIL: Tumor-infiltrating lymphocyte
TCGA: The Cancer Genome Atlas
TMA: Tissue microarray
IHC: Immunohistochemistry
IRS: Immune-reactivity score
TME: Tumor microenvironment

## References

[1] Siegel RL, Miller KD, Fuchs HE, Jemal A (2021). Cancer statistics, 2021. CA Cancer J Clin.

[2] Sung H, Ferlay J, Siegel RL, Laversanne M, Soerjomataram I, Jemal A (2021). Global cancer statistics 2020: GLOBOCAN estimates of incidence and mortality worldwide for 36 cancers in 185 countries. CA Cancer J Clin..

[3] Quaresma M, Coleman MP, Rachet B (2015). 40-year trends in an index of survival for all cancers combined and survival adjusted for age and sex for each cancer in England and Wales, 1971-2011: a population-based study. Lancet.

[4] Singh HM, Bailey P, Hubschmann D, Berger AK, Neoptolemos JP, Jager D (2020). Poly (ADP-ribose) polymerase inhibition in pancreatic cancer. Genes Chromosomes Cancer..

[5] Hu-Lieskovan S, Ribas A (2017). New combination strategies using programmed cell death 1/programmed cell death ligand 1 checkpoint inhibitors as a backbone. Cancer J.

[6] Aroldi F, Zaniboni A (2017). Immunotherapy for pancreatic cancer: present and future. Immunotherapy.

[7] Tang T, Huang X, Zhang G, Hong Z, Bai X, Liang T (2021). Advantages of targeting the tumor immune microenvironment over blocking immune checkpoint in cancer immunotherapy. Signal Transduct Target Ther.

[8] O'Reilly EM, Oh DY, Dhani N, Renouf DJ, Lee MA, Sun W (2019). Durvalumab with or without Tremelimumab for patients with metastatic pancreatic ductal adenocarcinoma: a phase 2 randomized clinical trial. JAMA Oncol..

[9] Cristescu R, Mogg R, Ayers M, Albright A, Murphy E, Yearley J (2018). Pan-tumor genomic biomarkers for PD-1 checkpoint blockade-based immunotherapy. Science.

[10] Fuchs CS, Doi T, Jang RW, Muro K, Satoh T, Machado M (2018). Safety and efficacy of Pembrolizumab monotherapy in patients with previously treated advanced gastric and gastroesophageal junction Cancer: phase 2 clinical KEYNOTE-059 trial. JAMA Oncol.

[11] Mei J, Xu J, Yang X, Gu D, Zhou W, Wang H (2021). A comparability study of natural and deglycosylated PD-L1 levels in lung cancer: evidence from immunohistochemical analysis. Mol Cancer.

[12] MacGregor HL, Ohashi PS (2017). Molecular pathways: evaluating the potential for B7-H4 as an Immunoregulatory target. Clin Cancer Res.

[13] Picarda E, Ohaegbulam KC, Zang X (2016). Molecular pathways: targeting B7-H3 (CD276) for human Cancer immunotherapy. Clin Cancer Res.

[14] Chen D, Li G, Ji C, Lu Q, Qi Y, Tang C (2020). Enhanced B7-H4 expression in gliomas with low PD-L1 expression identifies super-cold tumors. J Immunother Cancer.

[15] Cerami E, Gao J, Dogrusoz U, Gross BE, Sumer SO, Aksoy BA (2012). The cBio cancer genomics portal: an open platform for exploring multidimensional cancer genomics data. Cancer Discov.

[16] Mei J, Xu B, Hao L, Xiao Z, Liu Y, Yan T (2020). Overexpressed DAAM1 correlates with metastasis and predicts poor prognosis in breast cancer. Pathol Res Pract.

[17] Mei J, Yan T, Huang Y, Xia T, Chang F, Shen S (2019). A DAAM1 3′-UTR SNP mutation regulates breast cancer metastasis through affecting miR-208a-5p-DAAM1-RhoA axis. Cancer Cell Int.

[18] Yim J, Koh J, Kim S, Song SG, Ahn HK, Kim YA (2020). Effects of B7-H3 expression on tumour-infiltrating immune cells and clinicopathological characteristics in non-small-cell lung cancer. Eur J Cancer.

[19] Carvajal-Hausdorf D, Altan M, Velcheti V, Gettinger SN, Herbst RS, Rimm DL (2019). Expression and clinical significance of PD-L1, B7-H3, B7-H4 and TILs in human small cell lung Cancer (SCLC). J Immunother Cancer..

[20] Altan M, Pelekanou V, Schalper KA, Toki M, Gaule P, Syrigos K (2017). B7-H3 expression in NSCLC and its association with B7-H4, PD-L1 and tumor-infiltrating lymphocytes. Clin Cancer Res.

[21] Li YM, Yu JM, Liu ZY, Yang HJ, Tang J, Chen ZN (2019). Programmed death ligand 1 indicates pre-existing adaptive immune response by tumor-infiltrating CD8(+) T cells in non-small cell lung Cancer. Int J Mol Sci.

[22] Leung J, Suh WK (2014). The CD28-B7 family in anti-tumor immunity: emerging concepts in Cancer immunotherapy. Immune Netw.

[23] Ni L, Dong C (2017). New B7 family checkpoints in human cancers. Mol Cancer Ther.

[24] Guo L, Liu Z, Zhang Y, Quan Q, Huang L, Xu Y (2019). Association of increased B7 protein expression by infiltrating immune cells with progression of gastric carcinogenesis. Medicine (Baltimore).

[25] Qiu MJ, Xia Q, Chen YB, Fang XF, Li QT, Zhu LS (2021). The expression of three negative co-stimulatory B7 family molecules in small cell lung Cancer and their effect on prognosis. Front Oncol.

[26] Shen L, Qian Y, Wu W, Weng T, Wang FXC, Hong B (2017). B7-H4 is a prognostic biomarker for poor survival in patients with pancreatic cancer. Hum Pathol.

[27] Inamura K, Takazawa Y, Inoue Y, Yokouchi Y, Kobayashi M, Saiura A (2018). Tumor B7-H3 (CD276) expression and survival in pancreatic Cancer. J Clin Med.

[28] Gao HL, Liu L, Qi ZH, Xu HX, Wang WQ, Wu CT (2018). The clinicopathological and prognostic significance of PD-L1 expression in pancreatic cancer: a meta-analysis. Hepatobiliary Pancreat Dis Int.

[29] Cherif B, Triki H, Charfi S, Bouzidi L, Kridis WB, Khanfir A (2021). Immune checkpoint molecules B7-H6 and PD-L1 co-pattern the tumor inflammatory microenvironment in human breast cancer. Sci Rep.

[30] Ilie M, Long-Mira E, Bence C, Butori C, Lassalle S, Bouhlel L (2016). Comparative study of the PD-L1 status between surgically resected specimens and matched biopsies of NSCLC patients reveal major discordances: a potential issue for anti-PD-L1 therapeutic strategies. Ann Oncol.

[31] Patel SP, Kurzrock R (2015). PD-L1 expression as a predictive biomarker in Cancer immunotherapy. Mol Cancer Ther.

[32] Passiglia F, Bronte G, Bazan V, Natoli C, Rizzo S, Galvano A (2016). PD-L1 expression as predictive biomarker in patients with NSCLC: a pooled analysis. Oncotarget.

