# Supplementary material for: Clinical significance and correlation of PD-L1, B7-H3, B7-H4, and TILs in pancreatic cancer
Source: BMC Cancer. 2022 May 27;22:584. doi: 10.1186/s12885-022-09639-5 (PMC9137118; doi:10.1186/s12885-022-09639-5)
Supplement: Supplementary file 1 — Additional file 1: Fig. S1. Correlations of these three B7 molecules in PaCa tissues. (A) Correlation between PD-L1 and B7-H3 expression in PaCa tissues. (B) Correlation between PD-L1 and B7-H4 expression in PaCa tissues. (C) Correlation between B7-H3 and B7-H4 expression in PaCa tissues. [file 12885_2022_9639_MOESM1_ESM.docx]

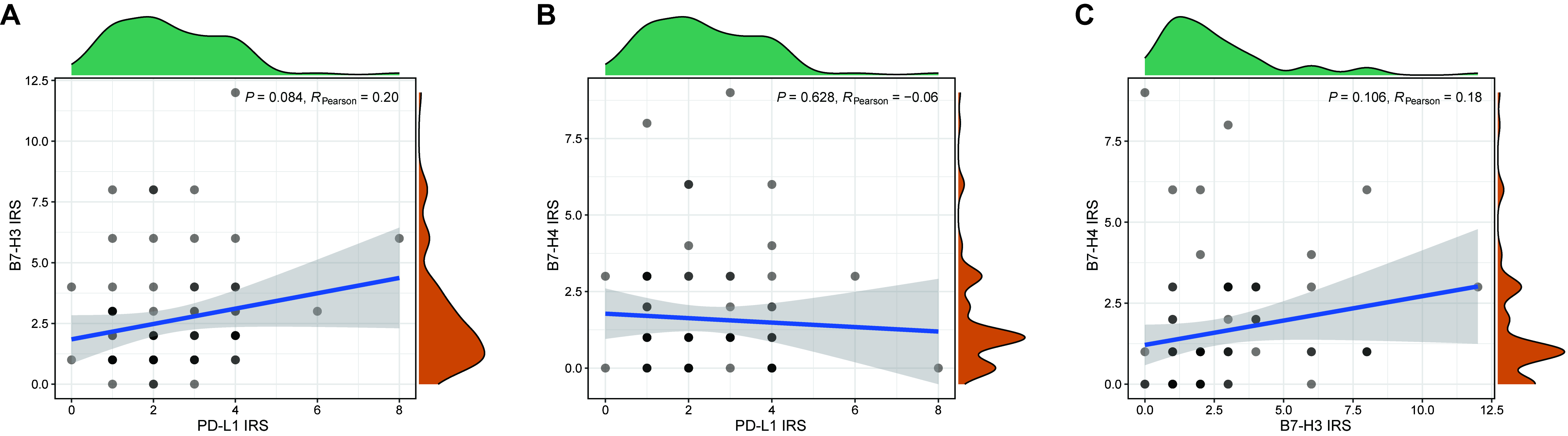


**Figure S1. Correlations of these three B7 molecules in PaCa tissues.**

(A) Correlation between PD-L1 and B7-H3 expression in PaCa tissues. (B) Correlation between PD-L1 and B7-H4 expression in PaCa tissues. (C) Correlation between B7-H3 and B7-H4 expression in PaCa tissues.
