# Supplementary material for: Clinical significance and correlation of PD-L1, B7-H3, B7-H4, and TILs in pancreatic cancer
Source: BMC Cancer. 2022 May 27;22:584. doi: 10.1186/s12885-022-09639-5 (PMC9137118; doi:10.1186/s12885-022-09639-5)
Supplement: Supplementary file 2 — Additional file 2: Fig. S2. Survival plots of these three B7 molecules in PaCa patients. (A) Prognostic value of PD-L1 mRNA expression in PaCa patients. (B) Prognostic value of B7-H3 mRNA expression in PaCa patients. (C) Prognostic value of B7-H4 mRNA expression in PaCa patients. Gene expression and survival data were obtained from UCSC Xena (https://xenabrowser.net/datapages/). [file 12885_2022_9639_MOESM2_ESM.docx]

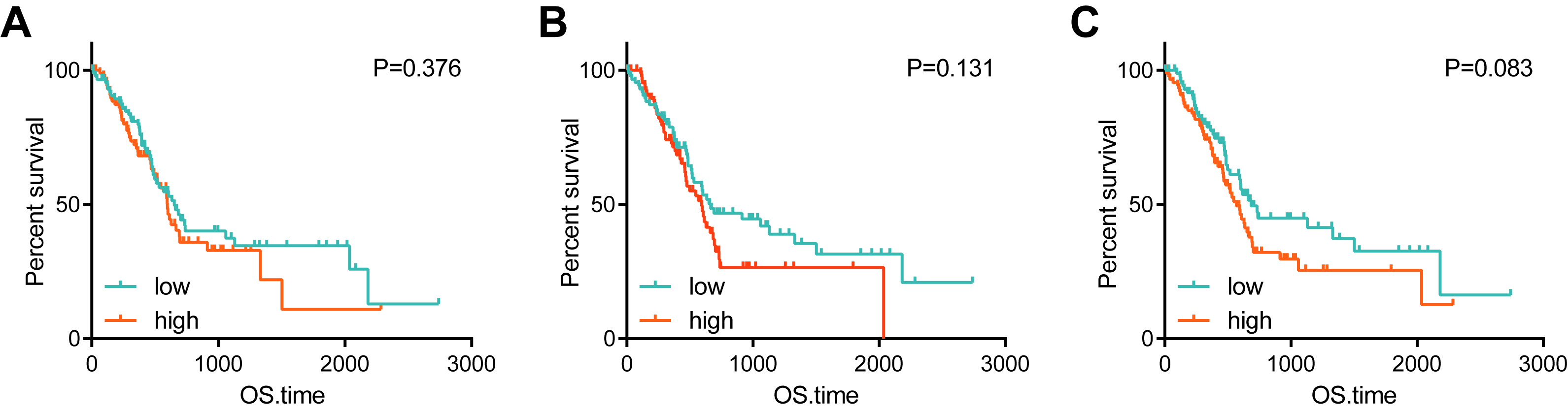
**Figure S2. Survival plots of these three B7 molecules in PaCa patients.**

(A) Prognostic value of *PD-L1* mRNA expression in PaCa patients. (B) Prognostic value of *B7-H3* mRNA expression in PaCa patients. (C) Prognostic value of *B7-H4* mRNA expression in PaCa patients. Gene expression and survival data were obtained from UCSC Xena (<https://xenabrowser.net/datapages/>).
