# Supplementary material for: Clinical significance and correlation of PD-L1, B7-H3, B7-H4, and TILs in pancreatic cancer
Source: BMC Cancer. 2022 May 27;22:584. doi: 10.1186/s12885-022-09639-5 (PMC9137118; doi:10.1186/s12885-022-09639-5)
Supplement: Supplementary file 3 — Additional file 3: Fig. S3. Correlations between PD-L1 & B7-H4 expression and CD8+ TILs infiltration in PaCa tissues (HPanA120Su02 cohort). (A) Correlation between PD-L1 expression and CD8+ TILs levels in PaCa tissues. (B) Correlation between B7-H4 expression and CD8+ TILs levels in PaCa tissues. [file 12885_2022_9639_MOESM3_ESM.docx]

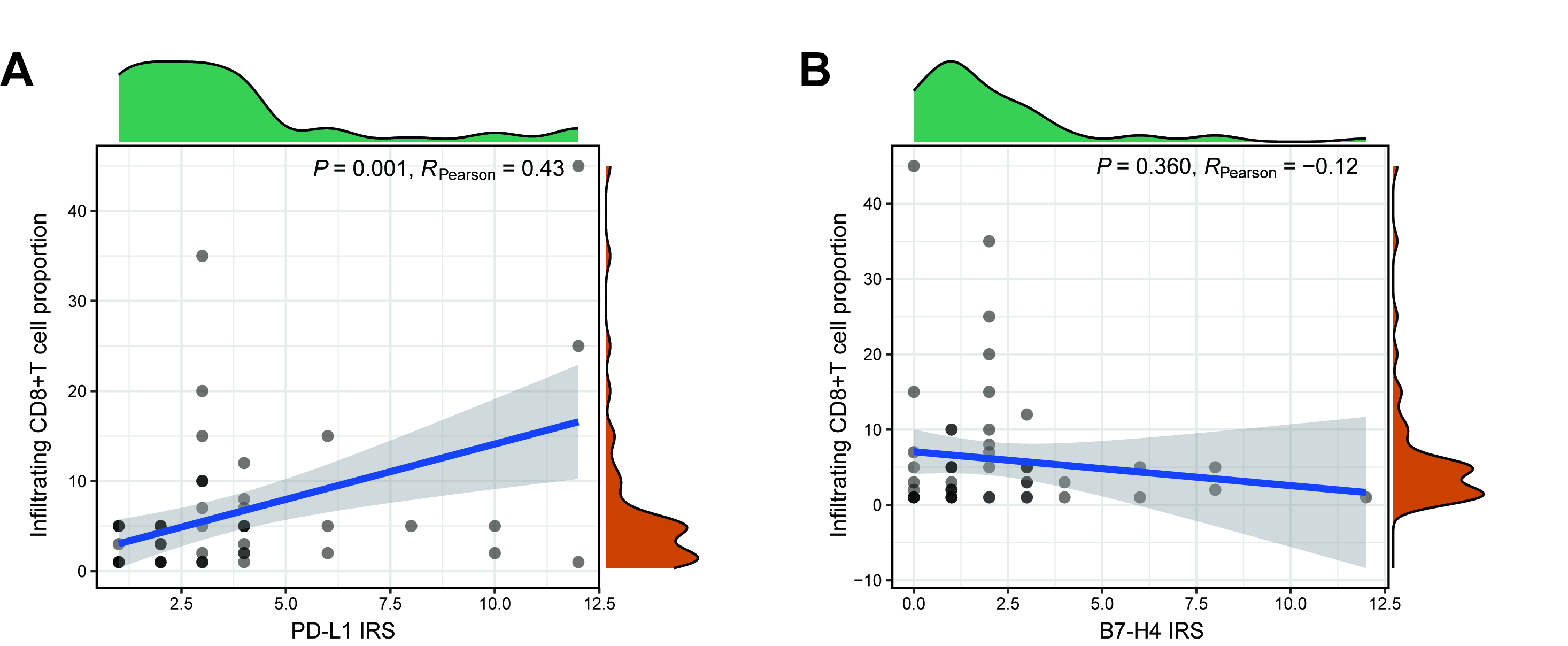


**Figure S3. Correlations between PD-L1 & B7-H4 expression and CD8+ TILs infiltration in PaCa tissues (HPanA120Su02 cohort).**

(A) Correlation between PD-L1 expression and CD8+ TILs levels in PaCa tissues. (B) Correlation between B7-H4 expression and CD8+ TILs levels in PaCa tissues.
